# Compressed air blast injury with palpebral, orbital, facial, cervical, and mediastinal emphysema through an eyelid laceration: a case report and review of literature

**DOI:** 10.1186/1471-2415-13-68

**Published:** 2013-11-07

**Authors:** Takahiro Hiraoka, Tomohiro Ogami, Fumiki Okamoto, Tetsuro Oshika

**Affiliations:** 1 Department of Ophthalmology, Faculty of Medicine, University of Tsukuba, 1-1-1 Tennoudai, Tsukuba, Ibaraki 305-8575, Japan

## Abstract

**Background:**

To the best of our knowledge, only 14 cases of orbital or periorbital compressed air injuries from air guns or hoses have been reported in the literature.

**Case presentation:**

A 30-year-old man was accidentally injured when a compressed air hose nozzle hit his right eye. The right half of his face was markedly swollen and a skin laceration near the right medial canthus was identified. A computed tomography scan showed subcutaneous and intraorbital emphysema around the right eye as well as cervical and mediastinal emphysema. He was prophylactically treated with systemic and topical antibiotics to prevent infection. All emphysemas had completely resolved 2 weeks after the injury.

**Conclusions:**

A review of all 15 cases (including ours) showed that all patients were male and that 6 of the 15 (40.0%) cases were related to industrial accidents. Although emphysema was restricted to the subconjunctival space in 2 (13.3%) cases, it spread to the orbit in the remaining 13 (86.7%) cases. Cervical and mediastinal emphysemas were found in 3 (20.0%) cases, and intracranial emphysema was confirmed in 6 (40.0%) cases. Prophylactic antibiotics were used in most cases and the prognosis was generally good in all but one patient, who developed optic atrophy and blindness.

## Background

Compressed air injuries (caused by air guns or hoses) most often occur in an industrial setting [1-7] and have been reported as a rare cause of orbital emphysema [1-6,8-13]. These injuries are generally accompanied by subconjunctival air bubbles [1-14], and sometimes involve intracranial emphysema [5,6,8,9,11,12]. These injuries can occasionally cause severe visual loss [8]. Only 14 cases of orbital or periorbital compressed air injuries from air guns or hoses have been sporadically reported in the literature (PubMed search using the search terms, “compressed air,” “emphysema,” “orbital,” “conjunctival,” “guns,” and “hoses.”); however, a systematic review of these cases is yet to be performed. We recently treated a patient with an orbital compressed air injury that resulted in emphysema, including mediastinal emphysema. Here, we report this case, and review the characteristics of the current case and the previously reported cases.

### Case presentation

**Figure 1:**
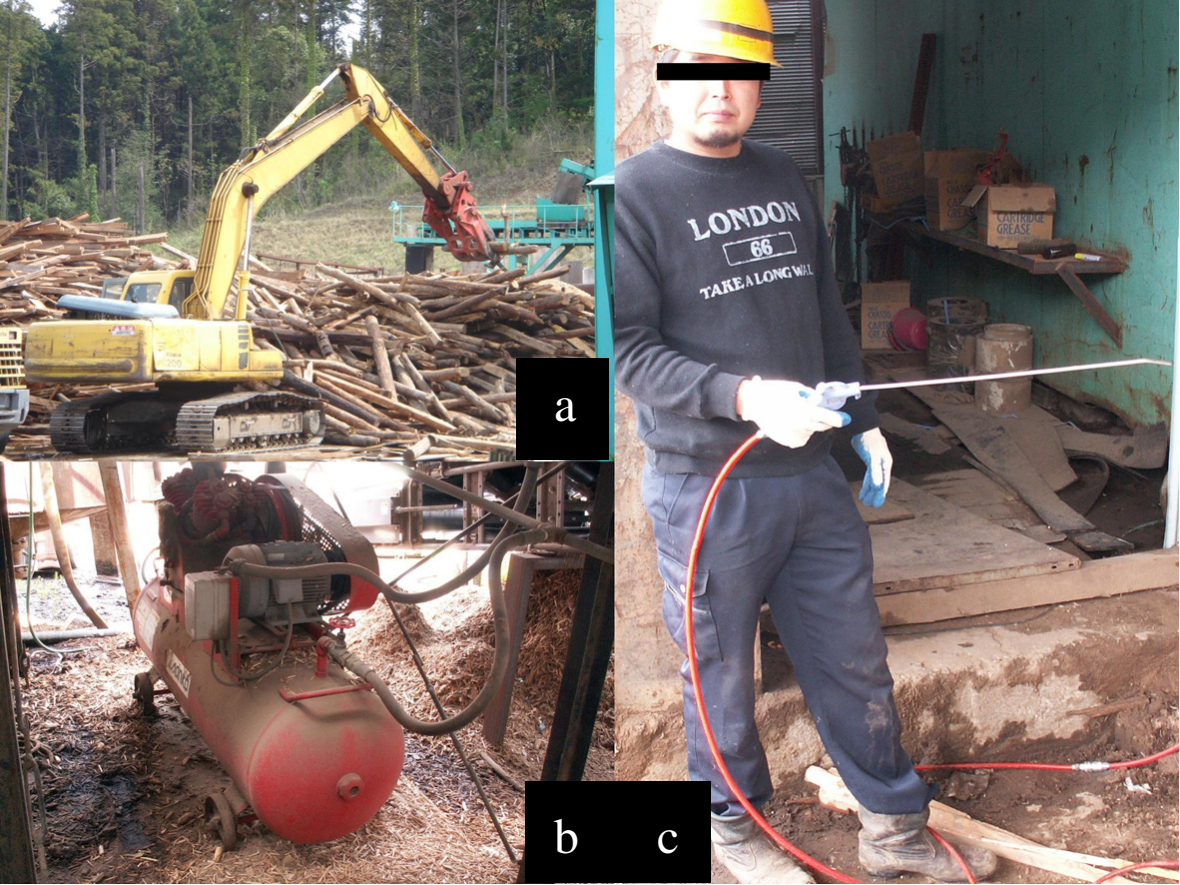
**Photographs of the sawdust factory and air compressor. (a)** The sawdust factory. **(b)** The main part of the air compressor (W-35, Fuji Compressor MFG. Co., Ltd). **(c)** The current patient holding the air hose and nozzle.

**Figure 2:**
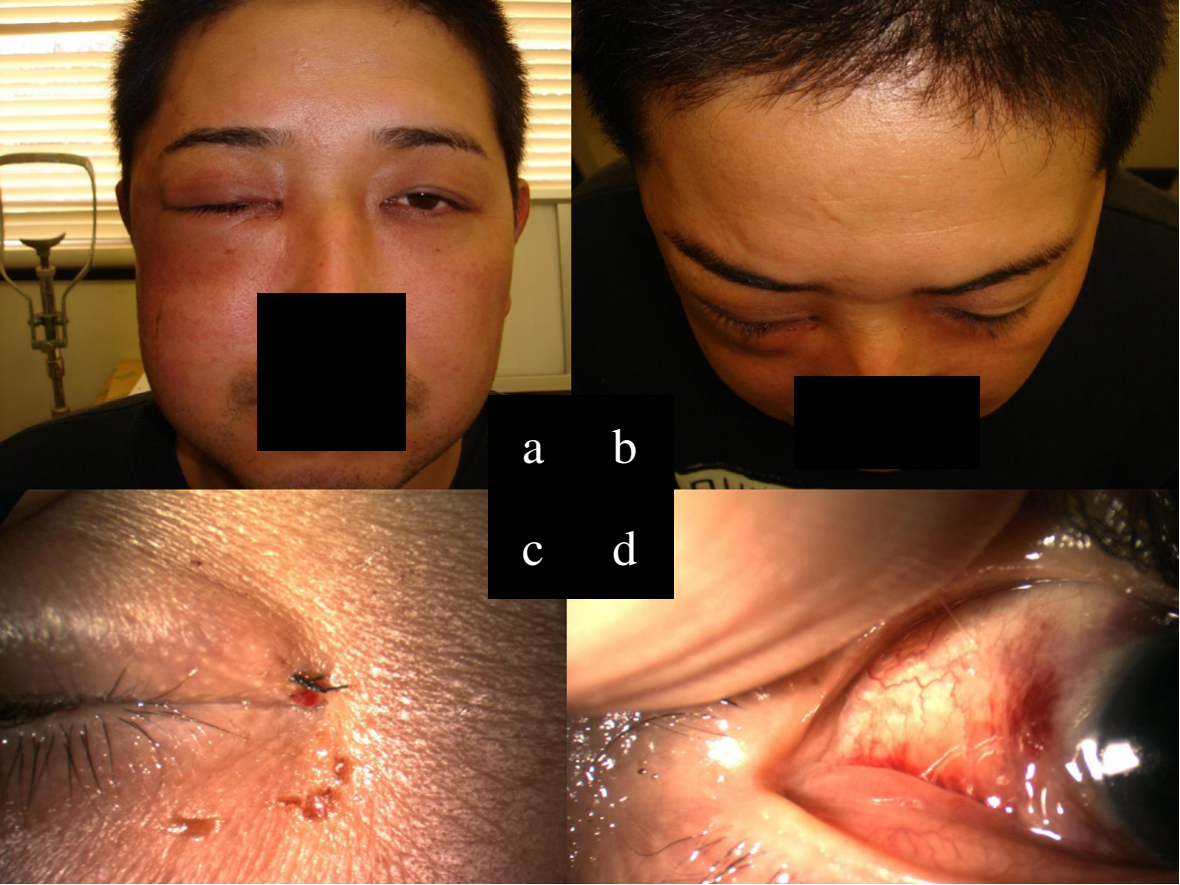
**Photographs of face and anterior segment of the eye. (a)** An external photograph demonstrates the swelling of the right eyelids and cheek. **(b)** Slight exophthalmos (3 mm) of the right eye was observed. **(c)** A small skin laceration (3 mm) near the right medial canthus was found. **(d)** Slit-lamp biomicroscopy showed subconjunctival emphysema and hemorrhage.

A 30-year-old male sawdust factory worker was using a compressed air jet with a 1/4 inch diameter nozzle on an air compressor hose (W-35, Fuji Compressor MFG. Co., Ltd), which expelled compressed air at approximately 170 psi. While cleaning wood particles from his clothes at the end of his working day (Figure 1a-c), he accidentally hit his right eyelid with the air hose nozzle. He noted immediate swelling of both eyelids, which was accompanied by pain, and was immediately brought to our ophthalmology service at the Tsukuba University Hospital. External examination revealed marked swelling of both the upper and lower right eyelids, along with the right half of the face. The palpebral fissure was narrowed (Figure 2a) and a slight exophthalmos (3 mm) of the right eye was observed (19 mm in the right eye, 16 mm in the left eye via Hertel exophthalmometer measurement, Figure 2b). A skin laceration near the right medial canthus (3 mm in length) was also identified on the eyelid (Figure 2c). Ocular movements in all directions were normal and decimal (Snellen in meters) best-corrected visual acuity (BCVA) was 0.6 (6/10) in the right eye and 1.5 (6/4) in the left eye. Intraocular pressure (IOP) was 15 mmHg in both eyes and slit-lamp biomicroscopy showed subconjunctival emphysema and hemorrhage (Figure 2d). The cornea was clear and had no epithelial defects. The anterior chamber was also clear and had no cells or flare. The crystalline lens was normal. Fundoscopy revealed a clear vitreous cavity a retina without abnormalities, including retinal breaks, detachment, or commotio retinae. The left eye and adnexa were completely normal.

**Figure 3:**
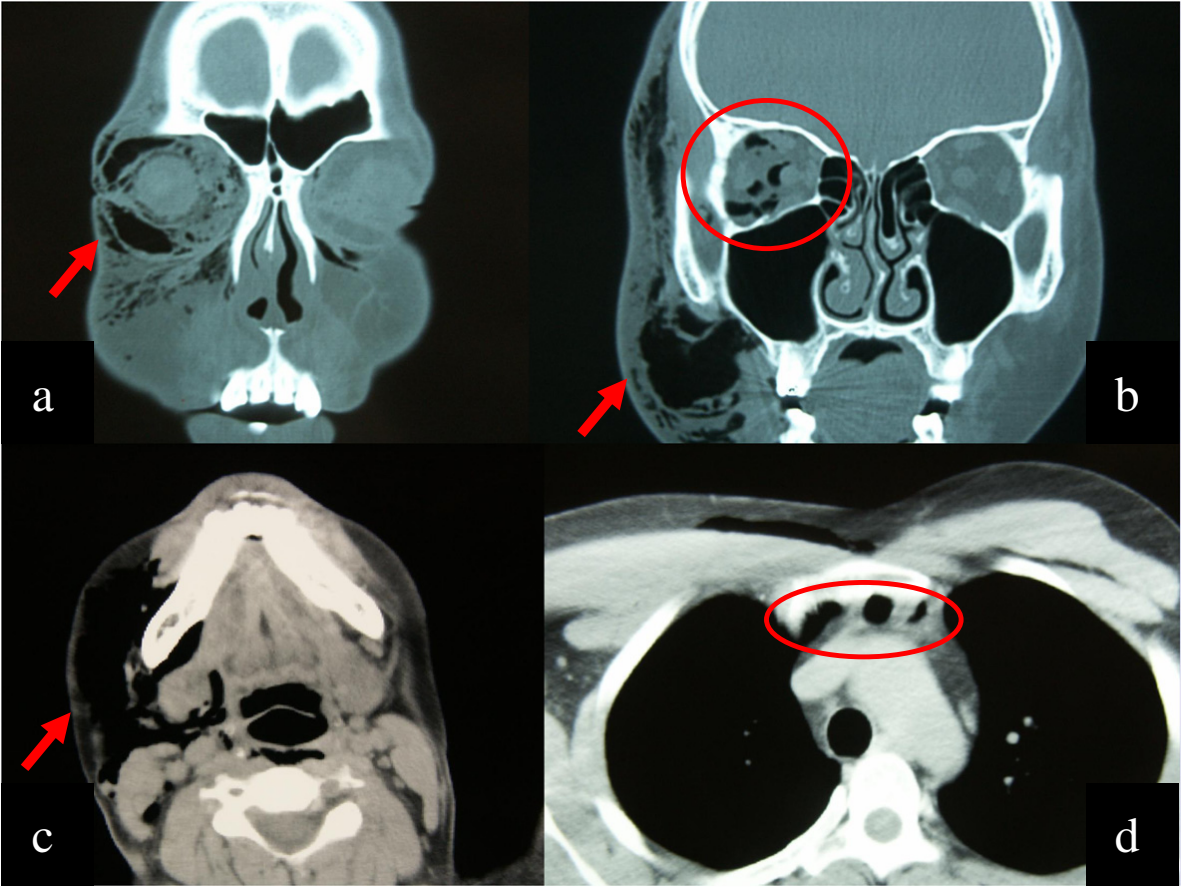
**An emergency computed tomography (CT) scan of the orbit, brain, neck, and chest. (a)** The CT demonstrated subcutaneous emphysema of the right eyelids (arrow). **(b)** Emphysema in the right orbit was observed (circle). Subcutaneous emphysema of cheek area was also found (arrow). There was no apparent air in the intracranial space and fracture of the orbital wall or floor. **(c)** The existence of subcutaneous air in the neck was also confirmed (arrow). **(d)** Mediastinal emphysema was also found (arrow).

An emergency computed tomography (CT) scan of the orbit, brain, neck, and chest showed subcutaneous eyelid emphysema, intraorbital emphysema, and right eye proptosis (Figure 3a,b). There were no apparent fractures of the orbital wall or floor, but the presence of subcutaneous air in the neck and emphysema in the mediastinum was confirmed (Figure 3c,d). It is thought that high-pressure air entered the eyelid skin and dissected subcutaneous tissues in the face and neck. It then presumably passed through the back surface of the sternum, eventually reaching the mediastinum. An internist was consulted to evaluate the neck and mediastinal emphysemas and help determine the best treatment. Because the patient had stable cardiopulmonary function, the internist recommended careful, close observation and prophylactic antibiotics. The patient was hospitalized for observation and was treated with systemic and topical antibiotics to prevent infection.

The lacerated wound of the eyelid was successfully closed with 7–0 nylon sutures (Figure 2c). Chest radiograpy 4 days after the injury showed no remaining air in the mediastinum. One week after injury, the BCVA returned to 1.0 (6/6) in the right eye, and the patient was discharged from the hospital. Although the exact cause of the decreased visual acuity at initial presentation was not determined, it is possible that the marked palpebral fissure narrowing and subconjunctival emphysema caused an unstable tear film. It may also have been that the intraorbital emphysema resulted in compression of the optic nerve. Two weeks following the injury, a CT scan was repeated and confirmed that all emphysemas had completely resolved. No ocular or systemic complications were identified at this time.

## Discussion

Although traumatic orbital emphysema is usually associated with fracturing of the orbital walls or paranasal sinuses [15], orbital emphysema without bone fractures often occurs in compressed air injuries. To the best of our knowledge, only 14 previous cases of orbital and/or subconjunctival emphysema from a compressed air injury have been reported. Here, we review these 14 cases, highlighting several interesting clinical features, and add an additional case to the literature (Table 1).

**Table 1:** Summary of clinical features of compressed air injuries

**Age, sex**	**Tool**	**Pressure of air jet (psi)**	**Relation to jobs**	**Entry site**	**Emphysema**		**Ocular motility**	**Ocular damage**	**Visual acuity**	**Treatment**	**Outcome**
**(Author)**					**Eye lid/conj/orbit**	**Other area**					
29 M	A compressed air hose (details unknown)	80	(+)	UN/L	(+)/(+)/(+)	(−)	Restricted in abduction and elevation	(−)	6/9	Systemic and topical antibiotics	Good
(Hitchings)											
6 M	A high pressure air hose (details unknown)	125 to 175	(−)	Conj/R	(+)/(+)/(+)	Face/mediastinum/intracranial space	UN	Mydriasis	No light perception	Prophylactic antibitics	Optic atrophy/blindness/ptosis
(King)											
16 M	A compressed air jet from a one fourth inch diameter nozzle on the hose to clean foam rubber particles moulds	90 to 100	(+)	Conj/R	(+)/(+)/(+)	(−)	Normal	Extensive keratitis/iritis/iris atrophy/IOP increase (25 mmHg)	20/40	Cycloplegic and steroid -antibiotic drops and tabs	Good
(Walsh)											
12 M	An air hose tip (details unknown)	UN	UN	Conj/L	(+)/(+)/(+)	Intracranial space	UN	Corneal abrasion/iritis	6/90	Intravenous penicillin	Good
(Koenig)											
55 M	A high-pressure air tube used in a garage	UN	(−)	Conj/L	(+)/(+)/(+)	(−)	Vertical limitation	Corneal edema/iritis/mydriasis/IOP increase (80 mmHg)	6/30	Incision of conj to release the trapped air/suture of conj wound	Good
(Teller)											
29 M	A compressed air tube to clean metal dust	50	(+)	Conj/L	(−)/(+)/(−)	(−)	UN	Metal foreign body in the cornea/IOP increase (28 mmHg)	Normal	Removal of the foreign body/incision of conj to release the air/antibiotic ointment	Good
(Biger)											
19 M	A compressed air hose (details unknown)	75	UN	Conj/R	(+)/(+)/(+)	Neck/mediastinum/intracranial space	Restricted (details UN)	Keratitis/iritis/commotio retinae/IOP increase (22 mmHg)	20/20	Ice packs/steroid, β-blocker, and antibiotic drops/systemic antibiotics	Good
(Lubniewski)											
34 M	A compressed air gun for automobile mechanic	100 to 120	(+)	Conj/R	(+)/(+)/(+)	(−)	Normal	Corneal abrasion	20/20	Erythromycin ointment	Good
(Stroh)											
47 M	An air compressor hose (details unknown)	120	UN	Conj/R	(+)/(+)/(+)	Face/intracranial space	Normal	Iritis/commotio retinae	20/100	Irrigation and debridement of conj wound/oral cephalexin/topical steroid and antibiotics	Good
(Williams)											
29 M	A compressed air gun to clean saws for timberyard workers	75	(+)	Conj/R	(+)/(+)/(+)	Face	Restricted in all directions	(−)	6/6	Systemic steroid/topical antibiotics	Good
(Caesar)											
24 M	A compressed air hose (details unknown)	UN	UN	Conj/L	(−)/(+)/(−)	(−)	Restricted in all directions	Keratitis/iritis	0.5	Lubricating drops and ointment	Good
(Kaiserman)											
22 M	A compressed air tube in a metal factory	UN	(+)	Conj/L	(+)/(+)/(+)	Face/intracranial space	Restricted in all directions	(−)	20/60	Suture of conj laceration/systemic and topical antibiotics	Good
(Yuksel)											
23 M	A compressed air gun to clean some tools	UN	UN	Conj/L	(+)/(+)/(+)	(−)	Restricted in all directions	Commotio retinae	20/30	Oral ampicillin and ibuprofen/topical ciprofloxacin eye ointment and flubiprofen eye drops	Good
(Mathew)											
49 M	A high-pressure compressed air jet to clean a tool in a workshop	UN	(+)	Conj/R	(+)/(+)/(+) fracture of the medial wall	Intracranial space	Diplopia in an upward gaze	Corneal erosion/iritis/commotio retinae	0.5	Suture of conj laceration/cooling with ice bags	Good
(Hwang)											
30 M	A compressed air jet from a one fourth inch diameter nozzle on the hose to clean wood particles in a sawdust factory	170	(+)	Eyelid/R	(+)/(+)/(+)	Face/neck/mediastinum	Normal	(−)	0.6	Laceration suture of eyelid/systemic and topical antibiotics	Good
Current case											

psi = pounds per square inch, conj = conjunctiva, VA = visual acuity, IOP = intraocular pressure, UN = unknown, R = right, L = left.

All 15 cases occurred in men, who were most commonly of working age. Six of the 15 (40.0%) cases occurred in industrial settings [1-7], and several cases occurred while patients were engaged in their hobbies [10,13]. The pressure of the compressed air causing these injuries ranged from 50 to175 psi. However, there appears to be no relationship between air pressure and injury severity.

In all cases, except for ours, the compressed air entry site was a conjunctival laceration. Because subconjunctival tissue is very loose, when compressed air enters through a conjunctival laceration it easily spreads to other regions, including the orbit. On the other hand, subcutaneous tissue is relatively tight, and thus it is more difficult for air to spread when it enters through a skin laceration. In our case, the power of the compressed air was quite strong (170 psi), which may be why the air was able to spread extensively to various regions, including the orbit, face, neck, and mediastinum.

Although emphysema was restricted to the subconjunctival space in 2 (13.3%) cases [7,14], it spread to the orbit in all the remaining 13 (86.7%) cases. Subcutaneous emphysema in the face was observed in 5 (33.3%) cases [4,5,8,10], cervical and mediastinal emphysemas were found in 3 (20.0%) cases [8,11], intracranial emphysema was confirmed in 6 (40.0%) cases [5,6,8,9,11,12], and ocular motility restriction and diplopia were found in 8 (53.3%) cases [1,4-6,10,11,13,14]. However, no apparent relationship between the location of emphysema and restriction of ocular movements was found.

Ocular damage was observed in 11 (73.3%) cases [2,3,6-14]. Corneal damage (e.g., keratitis, erosion, and edema) was observed in 7 (46.7%) cases [2,3,6,9-11,14], a corneal metal foreign body was found in 1 (6.7%) case [7], pupil dilation and iris atrophy were observed in 3 (20.0%) cases [2,8,10], and iritis was observed in 7 (46.7%) cases [2,9-12,14]. Furthermore, commotio retinae was found in 4 (26.7%) cases [6,11-13], elevated IOP was observed in 4 (26.7%) cases [2,7,10,11], and a visual acuity reduction (worse than 1.0 [6/6]) was observed in 11 (73.3%) cases [1,2,5,6,8-10,12-14].

Almost all patients were prophylactically treated with systemic and/or topical antibiotics to prevent infection. In some cases, topical and/or systemic steroids or local icing was also used to control inflammation. In cases that required surgical procedures, a conjunctival incision was made to release trapped air in 2 (13.3%) cases [7,10], conjunctival wound debridement was performed in 1 (6.7%) case [12], and laceration suturing was performed in 3 (20.0%) cases [5,6].

The outcome was generally good in these patients. The various emphysemas resolved and visual acuity returned to normal levels within several days to 1 month in all but one patient. Unfortunately, this patient developed optic atrophy, went blind, and had residual blepharoptosis [8]. Although the mechanism of optic nerve atrophy is not clear, it is believed that direct compression by the trapped air interrupted blood flow to the optic nerve [16]. Unfortunately, prognosis following this type of injury cannot be predicted by the severity or number of emphysemas or by the pressure in which the compressed air is expelled. However, physicians treating this type of injury should be aware of serious vision-threatening sequelae. On the basis of our review and observations, we recommend that protective goggles should be worn when working with compressed air tools.

## Conclusion

Here, we report a rare case of compressed air injury via an eyelid skin laceration. The patient showed not only subconjunctival and orbital emphysema but also facial, cervical, and mediastinal emphysemas. Fortunately, the air was absorbed within 2 weeks without severe permanent complications. By reviewing compressed air injuries reported in the literature, we showed that these types of accidents tend to occur in men, particularly those of working age. Compressed air injuries to the eye usually result in subconjunctival and orbital emphysemas, but may also lead to intracranial and/or mediastinal emphysemas in severe cases.

### Informed consent

Written informed consent was obtained from the patient for publication of this case report and any accompanying images. A copy of the written consent is available for review by the Editor of this journal.

## Competing interests

The authors declare that they have no competing interests.

## Authors’ contributions

TH: patient interaction and diagnosis, drafting of manuscript, final approval of manuscript. TO: patient interaction and diagnosis, final approval of manuscript. FO: final approval of manuscript. TO: critical revision and final approval of manuscript. All authors read and approved the final manuscript.

## Pre-publication history

The pre-publication history for this paper can be accessed here:

http://www.biomedcentral.com/1471-2415/13/68/prepub
